# *Slc11a1* gene polymorphism influences dextran sulfate sodium (DSS)-induced colitis in a murine model of acute inflammation

**DOI:** 10.1038/s41435-023-00199-7

**Published:** 2023-02-15

**Authors:** Stephane Tereza Queiroz de Andrade, Tamíris Isabela Guidugli, Andrea Borrego, Bridilla Luiza Colhado Rodrigues, Natália Coelho Couto de Azevedo Fernandes, Juliana Mariotti Guerra, Jean Gabriel de Sousa, Nancy Starobinas, José Ricardo Jensen, Wafa Hanna Koury Cabrera, Marcelo De Franco, Olga Martinez Ibañez, Solange Massa, Orlando Garcia Ribeiro

**Affiliations:** 1 Laboratório de Imunogenética, Instituto Butantan, São Paulo, Brazil; 2 Núcleo de Anatomia Patológica, Instituto Adolfo Lutz, São Paulo, Brazil

**Keywords:** Mucosal immunology, Immunogenetics

## Abstract

Ulcerative Colitis (UC) is an inflammatory disease characterized by colonic mucosal lesions associated with an increased risk of carcinogenesis. UC pathogenesis involves environmental and genetic factors. Genetic studies have indicated the association of gene variants coding for the divalent metal ion transporter SLC11A1 protein (formerly NRAMP1) with UC susceptibility in several animal species. Two mouse lines were genetically selected for high (AIRmax) or low (AIRmin) acute inflammatory responses (AIR). AIRmax is susceptible, and AIRmin is resistant to DSS-induced colitis and colon carcinogenesis. Furthermore, AIRmin mice present polymorphism of the *Slc11a1* gene. Here we investigated the possible modulating effect of the *Slc11a1 R* and *S* variants in DSS-induced colitis by using AIRmin mice homozygous for *Slc11a1* *R* (AIRmin^*RR*^) or *S* (AIRmin^*SS*^) alleles. We evaluated UC by the disease activity index (DAI), considering weight loss, diarrhea, blood in the anus or feces, cytokines, histopathology, and cell populations in the distal colon epithelium. AIRmin^*SS*^ mice have become susceptible to DSS effects, with higher DAI, IL6, G-CSF, and MCP-1 production and morphological and colon histopathological alterations than AIRmin^*RR*^ mice. The results point to a role of the *Slc11a1* S allele in DSS colitis induction in the genetic background of AIRmin mice.

## Introduction

Ulcerative Colitis (UC) is a chronic and relapsing inflammatory bowel disease (IBD) characterized by mucosal inflammation limited to the large intestine that predominantly affects the distal colon and rectum segments. The complete etiology of UC remains unknown, but it is accepted that genetic and environmental factors play essential roles in its development. The industrialization and westernization of lifestyles appear to be important environmental factors in UC. UC manifests with microbiota changes and rupturing of the epithelial barrier, leading to abnormal mucosal immune responses characterized by pro-inflammatory cytokine production, inflammatory cell activation, and decreased immunoregulation. Inflammation leads to mucosa destruction, causing diarrhea, bleeding, weight loss, abdominal pain, and other minor symptoms characteristic of the disease [1–3].

Different protocols of mucosal inflammation can experimentally reproduce the pathophysiology of UC; chemical induction by Dextran Sulfate Sodium (DSS) dissolved in drinking water is one of them [4]. The DSS model was first used with hamsters by Ohkusa’s group in 1985 and then with mice in Okayasu’s laboratory in 1990, with the animals manifesting clinical symptoms of diarrhea, occult blood, and severe rectal bleeding [5, 6]. The symptoms were caused by the direct action of DSS on the epithelial cell barrier, followed by the translocation of the microbiota from the intestinal lumen to the mucus layers, producing inflammatory reactions characterized by cell infiltration, mucin depletion, cryptitis, and crypt abscesses [7, 8].

As inflammatory cells, macrophages significantly contribute to the pathogenesis and chronicity of UC through cytokine production, the secretion of reactive oxygen species (ROS), and lymphocyte activation [9, 10]. Together with dendritic and B cells, macrophages play a critical role in intestinal homeostasis through the secretion of regulatory cytokines [11, 12]. Thus, the activities of different macrophage subtypes, and their balance between anti- and pro-inflammatory responses, are essential to the disease evolution [9].

In terms of genetic control, UC susceptibility must be controlled by multiple genes [9, 10]. Population studies have validated the association of UC with chromosomal loci regulating the epithelial barrier, apoptosis, autophagy, transcriptional regulation of interleukin production, T helper 1 and 17 cells (Th1 and Th17) differentiation, and the major histocompatibility complex (MHC) [13, 14].

Those associations with genes regulating immune responses correspond to disease characteristics caused by increased neutrophils, macrophages, and T lymphocytes within the colon mucosa. Cytokines produced by activated cells collaborate to cause local tissue destruction and the modulation of cell differentiation and proliferation, contributing to increased mucosal infiltration and an imbalance that favors the establishment of inflammatory processes [10]. Increased lymphocyte numbers may be associated with a Th2 lymphocyte response, as evidenced by increased IL-5 and IL-13 expression in colon tissue, spleen, and lymph nodes [15]. Additionally, T lymphocytes participate in pathogenic mechanisms by activating macrophages that contribute to high mucosa infiltration and the production of pro-inflammatory molecules, ROS, and reactive nitrogen intermediates [16, 17]. Macrophages are important contributors to UC pathogenesis, as they are present in the mucous membranes and are the primary source of inflammatory mediators.

The Solute carrier family 11 member A1 (*SLC11A1)* (formerly known as *Nramp1*) gene has been extensively investigated and encodes for a soluble bivalent transition metal transport protein that regulates macrophage activation. Meta-analysis studies have demonstrated the association of *SLC11A1* variants with autoimmune, infectious, and inflammatory diseases, including ulcerative colitis and Crohn’s disease [18–22].

The SLC11A1 protein exerts a range of pleiotropic effects during the activation of macrophages, including the induction of pro-inflammatory cytokines such as TNF-α, the regulation of MHC class II expression, and the production of ROS and reactive nitrogen intermediates [23].

*Slc11a1* mutations found in inbred mice substantially affect the growth rates of microorganisms during experimental infections by *Leishmania donovani*, some Mycobacterium species (*M. bovis, M. intracellulare, M. avium*, and *M. lepraemurium*), and *Salmonella enterica* serotype Typhimurium. Infection susceptibility in mice is associated with the replacement of the amino acid glycine by aspartic acid at position 169 (G169D). It results from a point mutation in the coding region of the fourth transmembrane domain of the gene. The normal WT allele (*R*) of the *Slc11a1* gene produces a functional protein in the phagolysosome membrane, while the mutated allele *(S*) produces a non-functional protein [24].

The G169D *Slc11a1* mutation causes deficiencies in macrophage activation and effector mechanisms (such as ROS and NO production), leading to intracellular pathogen infection susceptibility [25, 26].

A valuable and unique study using mice shows the role of *Slc11a1* mutation on the resistance or susceptibility to UC [27]. Studies of Japanese [28] and South African [28, 29] human populations have identified a possible association between the functional polymorphism of the *Slc11a1* gene and UC development, suggesting that the SLC11A1 ion transporter protein influences susceptibility to that disease. In addition to several genetic polymorphisms contributing to UC susceptibility, the presence of *Slc11a1* allele 3 (the promoter region among the seven microsatellite-mapped regions) responsible for the high expression of SLC11A1 protein was associated with increased pro-inflammatory responses and the activation of macrophage functions and could be used as an indicator of high risk for disease development in Caucasian individuals. In contrast, the same variant was associated with a low risk of UC among non-Caucasian populations [21]. These observations demonstrate the complexity of the genotype-phenotype relationship in their interaction with different and heterogeneous genetic backgrounds [27, 30].

Considering this, genetically heterogeneous mouse lines, besides inbred mice, can be used as an alternative model for UC studies. That approach allows the phenotypic evaluation of allelic combinations and may be more applicable to human populations. Accordingly, two mouse lines were selected based on acute inflammatory reactivity (AIR). These lines originated from a founding population generated by intercrossing eight inbred strains (A, DBA2, P, SWR, CBA, SJL, BALB/c, and C57BL/6) to ensure a great genetic variability, and the two lines, named AIRmax and AIRmin for maximum and minimum AIR respectively, were produced by several generations of bi-directional selective breeding based on cell counts and protein concentrations in local inflammatory exudates 24 h after s.c. injection of polyacrylamide beads (Biogel) [31].

After about 23 generations, the maximal separation of the two lines was achieved (limit of selection) and classical genetic analysis indicated that AIR regulation involves at least 11 Quantitative Trait Loci (QTL) harboring genes with alternative alleles with coherent effects for high or low inflammatory response [32–34]. AIRmax and AIRmin mice diverge widely in susceptibility or resistance to autoimmune diseases, chemically induced cancers in different organs, and bacterial and parasitic infections, demonstrating the impact and the biological significance of the modifications in AIR phenotypes of the two selected lines [33–35]. We also observed frequency disequilibrium of the *Slc11a1 R* and *S* gene alleles in AIRmax and AIRmin mice. The initial frequency of the *Slc11a1 S* allele was found to be 25% in the founding population (F0). However, that frequency became as high as 60% in AIRmin mice, and as low as 9% in AIRmax mice, after selection [36]. Accordingly, AIRmax mice are extremely resistant to *S*. Typhimurium infection, tolerating doses higher than 2 × 10^7^ bacteria, while the LD50 for AIRmin mice is 2 × 10^4^ bacteria [37].

In order to study the interaction of *Slc11a1* R and S alleles with the QTL that control AIR, we produced four sublines through genotypic assisted breeding, with the *Slc11a1 R* or *S* alleles fixed in homozygosity in AIRmax and AIRmin genetic backgrounds. Those lines named AIRmax^*RR*^, AIRmax^*SS*^, AIRmin^*RR*^, and AIRmin^*SS*^ maintained the phenotypes for maximum (AIRmax) and minimum (AIRmin) AIR, but a strong influence of the *S* allele was observed for susceptibility of the new sublines to *S*. Typhimurium infection and lipopolysaccharide (LPS) endotoxic shock, and to pristane induced arthritis (PIA) in both AIRmax and AIRmin genetic backgrounds [37].

Previous studies showed that AIRmax mice are susceptible and AIRmin resistant to DSS-induced colitis and colon carcinogenesis [33, 38]. Here, we investigated the influence of *Slc11a1* gene variants on the acute colitis phenotypes induced by DSS using AIRmin^*RR*^ and AIRmin^*SS*^ mouse lines derived from AIRmin-resistant mice as the experimental model. AIRmin^*RR*^ remained resistant, whereas AIRmin^*SS*^ became susceptible, suggesting a strong relationship between *the Slc11a1 S* allele and susceptibility to DSS-induced colitis.

## Materials and methods

### Mice

We used 3-month-old AIRmin male mice homozygous for the *R* or *S Slc11a1* alleles (AIRmin^*RR*^ and AIRmin^*SS*^). The animals were raised in the animal facilities of the Immunogenetics Laboratory at the Butantan Institute (São Paulo, Brazil), and the experiments were conducted according to the Butantan Institute Animal Ethics Committee (#1335/14).

### Colitis

We organized the two murine lines into two experimental groups to induce UC: the DSS-treated group AIRmin^*RR*^ and AIRmin^*SS*^ received 2.5% DSS (MP - Biomedicals, LLC, France) in distilled drinking water for seven consecutive days, while the control group AIRmin^*RR*^C and AIRmin^*SS*^C received only distilled water *ad libitum*. After seven days, we euthanized five mice per group from each strain for colon sampling and evaluation. The experiments were repeated 3 times.

### Disease activity index (DAI)

We first performed general clinical evaluations for colitis phenotype characterizations according to the following parameters: body weight changes, stool consistency, and presence of blood in the feces or the mouse anus, as indicated in Table 1 [39]. We weighed the animals before starting the experimental treatments and then checked for the presence or absence of diarrhea and rectal bleeding daily for seven days. We determined the DAI values during that period using the formula: DAI = bodyweight loss + diarrhea score + rectal bleeding score.

**Table 1:** Scores for DAI quantification, according to Wirtz 2007 [70].

Scores			
Scores	Weight loss	Diarrhea	Rectal bleeding
0	None	Absent	Absent
1	5–10%	Semi-pasty stools	Blood restricted to the anus in small amounts and/or in the stool
2	10–15%	Pasty stools	Blood in the stool in moderate amounts and/or beyond the anus
3	15–20%	Liquid stools	Blood in the stool and/or beyond the anus in abundant amounts
4	>20%	–	–

On the 7th day, the animals were euthanized to harvest colon segments, which we washed with saline and then measured their lengths. We cut the colon distal portions into three 1 cm long sections for histological analyses, cytokine determinations, and cell analyses.

### Histopathology

We performed histopathological analyses using the distal portions of the colon samples that had been fixed in 10% paraformaldehyde for 24 h and stored in 70% ethanol. The tissue was subsequently embedded in paraffin, and 5 μm thick sections were prepared and stained with hematoxylin and eosin (HE). The histopathological blinded analysis were performed by pathologists at the Pathology Department of the Adolfo Lutz Institute (São Paulo, Brazil) following the classification of the histological parameters [40] (Table 2).

**Table 2:** Classification of the histological parameters.

Scores								
Category	Inflammatory infiltrate			Tissue Architecture			Epithelial Alteration	
Scores	Severity	Extension	Cellular infiltrate	Erosion	Tissue Granulation	Submucosa Edema	Hyperplasia	Loss/Necrosis
1	Minimum < 10%	Mucosa	lymphohistiocytic	1	1	1	Minimum < 25%	Minimum < 25%
2	Discreet 10–25%	Mucosa and Submucosa	Mixed	2	2	2	Discreet 25–35%	Discreet 25–35%
3	Moderate 26–50%	Transmural	Heterophilic	3	3	3	Moderate 36–50%	Moderate 36–50%
4	Marked > 51%	-		4	4	4	Marked > 51%	Marked > 51%

### Protein extraction

To evaluate the production of pro- and anti-inflammatory mediators, we homogenized the colon sections and extracted their proteins using the TissueLyser LT (Qiagen) protocol. Accordingly, we transferred 30 mg of distal colon fragments to 2 mL microcentrifuge tubes containing a stainless steel bead (5 mm in diameter) for pre-cooling on dry ice for 15 min. We then transferred the tubes to a TissueLyser LT adapter (maintained at 15–25 °C) for incubation at room temperature for 2 min in 1 mL RIPA buffer (Sigma). The TissueLyser LT was operated for 4 min at 50 Hz for complete tissue homogenization, and the supernatant was stored at −80 °C until use.

### Multiplex assays

We evaluated the production of IL-1β, IL-4, IL-6, IL-10, IL-17, IFN-γ, and TNF-α cytokines, MCP-1, and MIP-1α chemokines, and locally produced G-CSF, GM-CSF, and M-CSF growth factors using distal colon lysates in a Luminex assay, following the instructions of the MCYTOMAG-70K kit (Merk/Millipore); the data were analyzed using Analyst software (Luminex).

### Gene expression (RT-PCR)

Total RNA was isolated from approximately 30 mg of distal colon fragments using the TissueLyser LT (Qiagen) with 1 mL of lysis buffer and operated for 4 min at 50 Hz. The samples were then applied to an RNAspin mini-filter unit for RNA purification according to the manufacturer’s protocol (GE Healthcare UK Limited Kit). RNA quantification was measured in a NanoVue spectrophotometer (GE Healthcare Lifesciences), and its degradation level was assessed on a denaturing agarose gel. From the extracted RNA, 1 μg was used to synthesize cDNA by reverse transcription using First Strand cDNA Synthesis Kit (Roche, Basel, Switzerland). Five μL of the cDNA was used in 1:10 dilution, then 10 µL of Fast SYBR Green Master Mix (Life Technologies, Foster City, USA) and 300 μM of the primers described below were added in a final reaction volume of 20 µL. The assays were carried out in a StepOnePlus real-time detector. The expression levels of several reference genes (Rps29, HPRT, Ppia, and B2m) were analyzed for stability with the geNorm VBA applet [41], and the combination of RPS29 (Ribosomal Protein S29), Ppia (Cyclophylline) was determined as the most stable. Primer pairs of the genes *Il1β*, *Il6*, *Il10*, *Il17*, *Tnfα*, *Infγ*, *Mcp1*, *Gmcsf*, and *Mip1α* are listed in Table 3. The Ct values were calculated, and the relative expression was determined as 2^−∆∆Ct^ [42, 43].

**Table 3:** Primer pairs used in qRT-PCR assays.

Gene	Forward primer (5′-3′)	Reverse primer (5′-3′)
*Ppia*	AGCGTTTTGGGTCCAGGAAT	AAATGCCCGCAAGTCAAAAG
*Rps29*	TCTACTGGAGTCACCCACGGAAGT	GTCAGTCGAATCCATTCAAGGTCGC
*Il1b*	TTGACGGACCCCAAAAGATG	AGAAGGTGCTCATGTCCTGA
*Il6*	GTTCTCTGGGAAATCGTGGA	TGTACTCCAGGTAGCTATGG
*Il10*	GCTGGACAACATACTGCTAACC	CCCAAGTAACCCTTAAAGTCCTG
*Il17*	TGAACCTCCTGGATGACATG	GTGTTTCACAGTCCGTTTCC
*Tnf*∝	TCTCATCAGTTCTATGGCCC	GGGAGTAGACAAGGTACAAC
*Ifnγ*	GCTCTGAGACAATGAACGCT	AAAGAGATAATCTGGCTCTGC
*Mcp1*	GCCTGCTGTTCACAGTTGC	TCATTGGGATCATCTTCGTG
*Gmcsf*	TGAACTCCTGGATGACATG	GTGTTTCACAGTCCGTTTCC
*Mip1*∝	GGTCTCCACCACTGCCCTTCG	GGTGGCAGGAATGTTCGGCTC

### Flow cytometry

Fresh cells were isolated from the distal colon segment incubating in digest solution (1 mg/mL collagenase and 1 U/mL DNase I in RPMI supplemented with 5% fetal calf serum, 1% L-glutamine, 1% penicillin-streptomycin, and 10 mM HEPES) for 40 min, at 37 °C and passed through a 100 µm strainer, and purified using 35% Percoll. Following centrifugation at 400 x g, the pellet was suspended in 1 mL of RPMI medium and counted in a Malassez hemocytometer chamber [44]. We labeled a 100 μL aliquot of cell suspension (1 × 10^5^ cells/mL) with specific rat antibodies against CD4 (clone RM4-5), CD8_a_ (clone 53-6,7), GR1 (clone RB6-8C5), CD11b (clone M1/70), F4/80 (clone BM8), MHC II (clone M5/114.15.2), and IL-10 (clone SXC-1) murine molecules. We used phycoerythrin-labeled (PE)- or fluorescein isothiocyanate (FITC)-labeled mouse IgG2b (clone: A95-1) as control isotypes. All antibodies were purchased from BD Biosciences (Pharmigen Franklin Lakes, NJ, USA). We recorded 50,000 events using a FACSCanto II flow cytometer (Becton Dickinson) and analyzed them using FlowJo software (Tree Star).The cell concentrations were calculated by multiplying the total number of viable cells obtained per mL (counted using Trypan blue exclusion) by the percent of the viable cells gate during flow cytometric analysis. In these experiments, we used five DSS-treated mice and three control mice in each group.

### Statistical analysis

We used a one-way analysis of variance (ANOVA) to statistically determine significant differences between the mean values of the groups; *p* < 0.05 was considered significant. Data were analyzed using GraphPadPrism 4.0 software (GraphPad Software, San Diego, CA, USA).

## Results

### Colitis phenotype

In the first series of experiments, we compared UC severity between AIRmin^*RR*^ and AIRmin^*SS*^ mice during 2.5% DSS treatment by determining the disease activity index (DAI). DAI evaluations were performed using the parameters described in Table 1 and recorded for seven consecutive days.

**Fig. 1:**
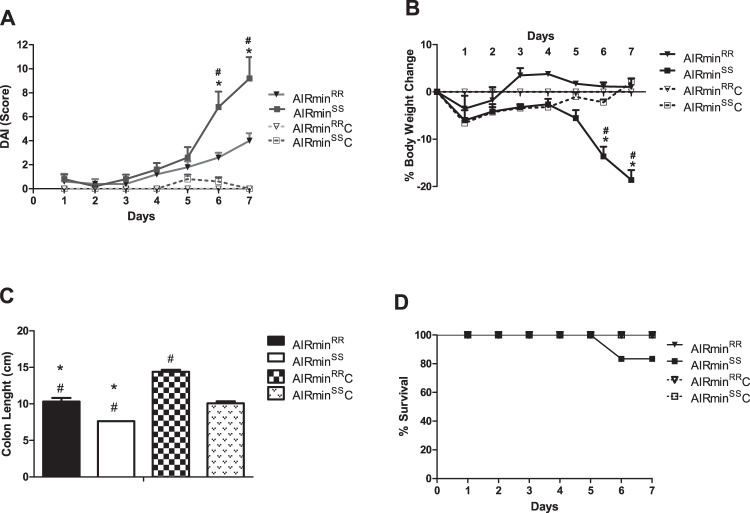
Colitis phenotypes. Groups of 5 mice of each line were evaluated concerning DAI evolution (**A**) percentage weight loss (**B**) colon length (**C**) and survival (**D**) after seven days of access to pure water (AIRmin^*RR*^C and AIRmin^*SS*^C) or 2.5% DSS (AIRmin^*RR*^ and AIRmin^*SS*^). The values shown are the means ± SEM from 3 different experiments with five animals in each group. Comparisons between DSS-treated AIRmin^*SS*^ and AIRmin^*RR*^ (#), or between DSS and water-treated mice (_*_) using ANOVA, *p* < 0.05.

As shown in Fig. 1A, the AIRmin^*SS*^ group showed the highest DAI levels. Those mice symptoms worsened starting on the 5th day, reaching high levels on the 6th and 7th days, with substantial intestinal bleeding. They had lost almost 20% of their body weight by the 7th day (Fig. 1B). AIRmin^*RR*^ mice displayed milder symptoms. Another characteristic evaluated was the change in colon length (considered from the distal [straight] region to the beginning of the cecum). DSS-treated AIRmin^*SS*^ mice showed a greater decrease in colon length, demonstrating the severity of UC in this line and confirming the DAI phenotype data (Fig. 1C). Only one AIRmin^*SS*^ mouse succumbed to exposure to DSS (Fig. 1D).

### Histological analysis

We performed histological analyses of the colons on the 7th day of treatment with 2.5% DSS to evaluate the degrees of tissue alteration (using a fragment of the distal colon segment to prepare slides for analysis). The analyses followed the criteria suggested by Erben et al. (2014), who established scores to represent the levels of inflammation, tissue architecture, and alteration of the epithelium, as presented in Table 2.

**Fig. 2:**
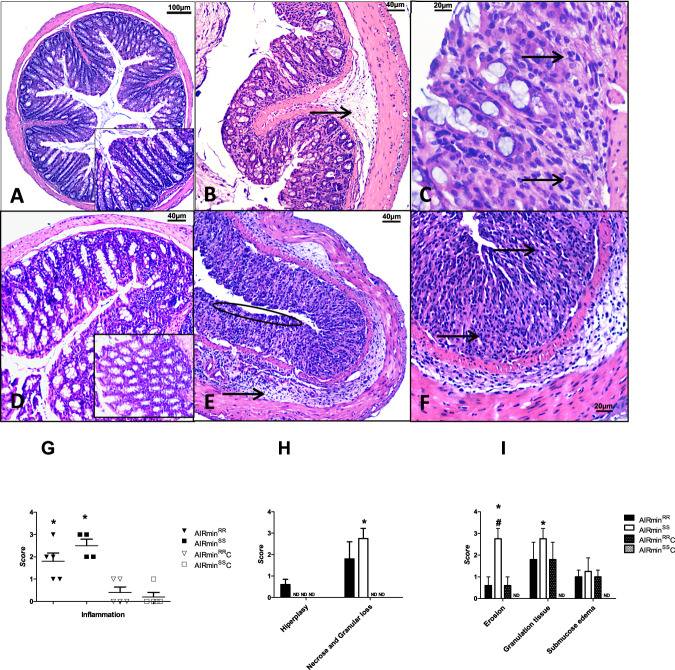
Histopathological analysis. Representative photomicrographs of distal colon sections were obtained from AIRmin^*RR*^C and AIRmin^*SS*^C mice with access to pure water (**A**, **D**) or from AIRmin^*RR*^ and AIRmin^*SS*^ with access to 2.5% DSS for seven consecutive days (**B**, **C**, **E**, and **F**). **A** (magnification of 40x) and **D** (magnification of 100x) with typical colonic epithelial cells, better shown in the inset, and none or rare inflammatory cells. **B** AIRmin^*RR*^ colon, with discreet loss of epithelial cells and discreet tissue granulation. The arrow indicates submucosa edema associated with mild inflammatory infiltrate (magnification of 100x). **C** AIRmin^*RR*^ colon, with epithelial loss and moderate inflammatory infiltrate, indicated by arrows (magnification of 200x). **E** AIRmin^*SS*^ colon, with marked loss of glandular cells and substitution by granulation tissue. The circle indicates erosion, and the arrow indicates moderate submucosa edema associated with moderate inflammation (magnification of 100x). **F** AIRmin^*SS*^ colon, the loss of epithelial cells in higher magnification, with arrows pointing to the granulation tissue and mucosal inflammation (magnification of 200x). Histopathological characteristics by scores of the Intensity of Inflammatory Reaction (**G**) tissue architecture (**H**) and epithelial (**I**) alterations. The values shown are the means ± SEM of 5 animals per group. Comparisons between DSS-treated and control mice (_*_) or between AIRmin^*SS*^ and AIRmin^*RR*^ mice (#) by ANOVA, *p* < 0.05. ND = not detected.

Figure 2 shows representative photomicrographs of untreated (A, D) or DSS-treated (B, C, E, and F) groups. Analyses of the scores (represented by medians) showed differences concerning inflammatory infiltrates, tissue architecture destruction, and epithelial changes. Although AIRmin^*RR*^ exhibited mixed inflammatory infiltrates and variable degrees of epithelial lesions (Fig. 2BC), AIRmin^*SS*^ mice were more affected – showing highly inflammatory infiltrates and severely compromised colon architectures with extensive epithelial barrier destruction (arrow) (Fig. 2E–F). We also show the means of the parameters considered in the histological analysis: inflammation (Fig. 2G) and tissue and epithelium changes (Fig. 2H and I). The analysis of those parameters showed that although AIRmin^*RR*^ demonstrated equivalent increases in inflammation, with scores near 2, the AIRmin^*SS*^ animals had more tissue or epithelial architecture alterations, especially erosion, demonstrating their higher sensitivity to DSS.

### Cell populations

**Fig. 3:**
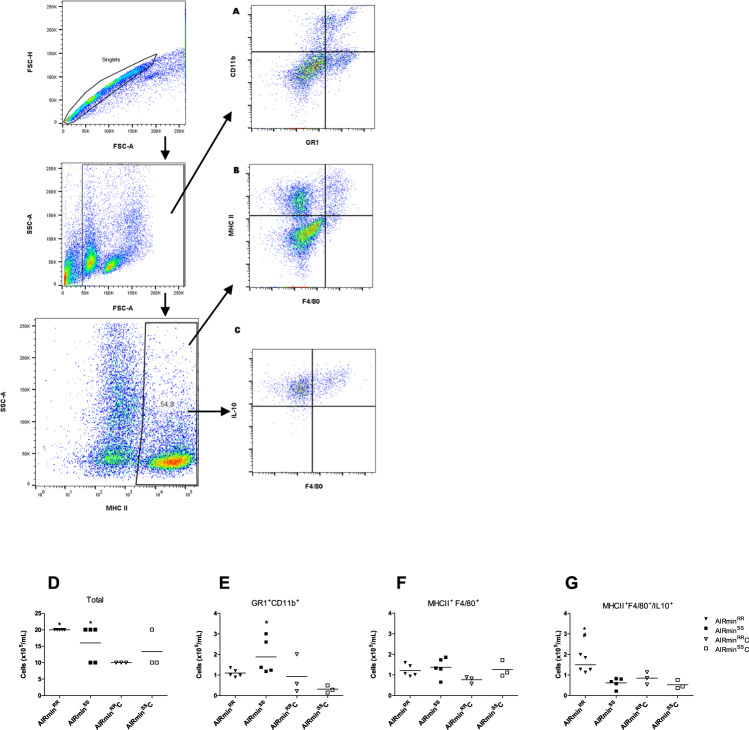
Flow Cytometry analyses of cellular infiltrate into distal colon segments from 2.5% DSS treated (*n* = 5) AIRmin^*RR*^ and AIRmin^*SS*^ or control AIRmin^*RR*^C and AIRmin^*SS*^C mice (*n* = 3). Representative cytograms of Gr1^+^CD11b^+^ (**A**) MHCII^+^F4/80^+^ (**B**) MHCII^+^F4/80^+^IL10^+^ (**C**) Graphs show unselected (**D**) Gr1^+^CD11b^+^ (**E**) MHCII^+^F4/80^+^ (**F**) and MHCII^+^F4/80^+^IL10^+^ cell concentrations with the indication of mean values (**G**) Each cell concentration value was calculated based on the percentage obtained from the respective cytograms. Comparisons between treated and control mice (_*_) or between AIRmin^*RR*^ and AIRmin^*SS*^ (#) mice using ANOVA, *p* < 0.05.

The cell populations present in the colon were obtained by digestion of the segments by collagenase on the 7^th^ day of treatment from the distal segments of the colons of mice treated with 2.5% DSS and from control mice. Fig. 3D shows the total number of cells per mL in each group obtained from the colon; cell numbers ranged from 2 to 3 × 10^6^ cells/mL.

Phenotyping of the different cell populations was performed by the use of specific antibodies using the labeling strategy of Mowat and Bain (2011) [45, 46] for granulocytes (CD11b^+^GR1^+^) macrophages (MHCII^+^/F4/80^+^), and macrophages with regulatory functions (MHCII^+^F4/80^+^IL-10^+^). AIRmin^*SS*^ mice exhibited higher levels of Gr1^+^CD11b^+^ infiltrated inflammatory cells (Fig. 3E) than AIRmin^*RR*^, but there was no increase in MHCII^+^/F4/80^+^ macrophages in both lines. (Fig. 3F). On the other side, the macrophage population with regulatory functions (MHCII^+^F4/80^+^IL-10^+^) increased in DSS-treated AIRmin^*RR*^ mice but not in the AIRmin^*SS*^ group, which maintained basal levels (Fig. 3G). We analyzed the CD4^+^ and CD8^+^ T cell populations but did not observe significant differences between the two mouse lines under our experimental conditions (Supplementary Fig. S1).

### Cytokines

**Fig. 4:**
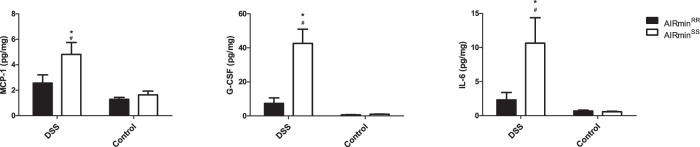
IL-6 cytokine, MCP-1 chemokine, and G-CSF growth factor concentrations in distal colon lysates of mice with access to 2.5% DSS (AIRmin^*RR*^ and AIRmin^*SS*^) or pure water (AIRmin^*RR*^C and AIRmin^*SS*^*C*) for seven days. Values are shown as means ± SEM of 5 animals per group. Comparisons between treated and control mice (_*_), or between AIRmin^*RR*^ and AIRmin^*SS*^ (#) mice using ANOVA, *p* < 0.05.

We determined the levels of IL-1β, IL-6, IFNγ, TNFα, IL-17, and IL-10 cytokines, MCP-1 and MIP-1α chemokines, and G-CSF, GM-CSF, and M-CSF growth factors by multiplex assays of the lysates obtained from samples of the distal colon fragments from DSS-treated and control mice. Considering all three groups of mediators, we found differences only in IL6, G-CSF, and MCP-1 factors, with the highest levels observed in DSS-treated AIRmin^*SS*^ mice (Fig. 4).

Comparisons between treated and control mice (*) and interlines (#) using ANOVA, *p* < 0.05.

### RT-PCR gene expression analysis

**Fig. 5:**
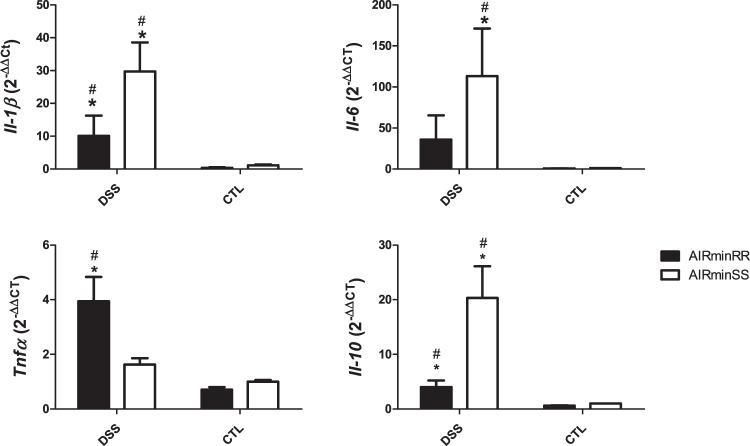
Cytokine and Chemokine gene expression in distal colon cells of 2.5% DSS-treated AIRmin^*RR*^ and AIRmin^*SS*^ or AIRmin^*RR*^C and AIRmin^*SS*^C as control mice. Values are shown as means ± SEM of 5 animals per group. Comparisons between treated and control mice (_*_) or between AIRmin^*RR*^ and AIRmin^*SS*^ (#) using ANOVA, *p* < 0.05. Data are the normalized (2^−ΔΔCt^) expression of each sample.

The genes selected for real-time PCR studies were based on multiplex data to confirm the results observed for cytokines, chemokines, and growth factors on the 7th day of DSS supply. Unlike *Tnfa*, the *Il1β*, *Il6*, and *Il10* gene expressions were significantly higher in AIRmin^*SS*^ mice than in AIRmin^*RR*^ mice (Fig. 5).

## Discussion

The etiology of ulcerative colitis has not yet been fully elucidated but can be characterized by an inflammatory process of the intestinal mucosa involving cytokines and various types of cells, including dendritic cells, macrophages, T helper cells, regulatory T cells, and natural killer T cells [47]. As it is a complex multifactorial disease, its genetic regulation must involve various genes. Many candidate genes have been described as regulating UC, but their actual roles in the disease have not been extensively examined. In the present study, we sought to evaluate the involvement of the *Slc11a1* gene in experimental DSS-induced UC development.

The *Slc11a1* gene encodes a Fe^+^ and Mn^+^ transporter protein in leukocyte populations (especially in macrophages and monocytes) that is important in fighting bacterial infections. Jiang et al. (2009) first experimentally demonstrated an association of this gene with DSS-induced UC. The authors used a pair of congenic mice carrying the WT or mutant *Slc11a1* alleles onto a C57BL/10ScSn background. These mice are congenic with *Slc11a1* mutant B10 mice except for the 10 Mb interval on chromosome 1 that carries the *Slc11a1* gene. The presence of a mutation that caused the loss of protein function improved colitis symptoms when the parameters of weight loss and reduction of colon length were evaluated [27].

Our group studied DSS (40 kDa)-induced UC in genetically heterogeneous AIRmax and AIRmin mice maintained under Specific Pathogen-Free conditions [33]. We evaluate parameters related to UC (including weight loss, stool consistency, and bleeding), composing the DAI proposed by [48]. AIRmax mice were found to show higher UC severity than AIRmin mice after 2.5% DSS treatment. Considering the central inflammatory component in UC development, those results followed the respective AIRmax and AIRmin phenotypes of high and low acute inflammatory responses, respectively [33, 48].

Due to the genetic polymorphism of the *Slc11a1* gene found in those lines [36] and aiming to study the effect of this polymorphism in DSS-induced UC, we essayed the protocol in AIRmin mouse lines homozygous for the *R* (resistance) and *S* (susceptibility) alleles of the *Slc11a1* gene.

In contrast with the results published by Jiang [27], in our study in which the mutated allele is expressed in the AIRmin heterogeneous genetic background, AIRmin^*SS*^ mice showed higher sensitivity to DSS than the AIRmin^*RR*^ bearing the WT allele, with significant increases in DAI (characterized by diarrhea, bleeding, and marked weight losses), colon alterations and cytokines production. Our results thus demonstrated a relationship between the presence of the *Slc11a1*^*SS*^ allele and greater reactivity to DSS by AIRmin^*ss*^ mice. A similar situation was described among different human populations where a relationship between SLC11A1 polymorphisms and susceptibility to UC was especially identified in Caucasian population [21].

We conducted histological analyses to determine the degrees of tissue damage caused by DSS and correlated them with clinical symptoms. Inflammatory infiltrates were present in the two groups, although with different effects on the intestinal mucosa. We considered two categories in evaluating the damage caused by the treatment: (i) tissue architectural damage represented by erosion, tissue granulation, and submucosal edema; and (ii) epithelial modifications, considering hyperplasia and necrosis [40].

AIRmin^*SS*^ mice exhibited the most severe lesions, characterized by tissue architecture damage due to severe erosion, tissue granulation, and epithelial changes with extensive glandular necrosis/loss. Those alterations are commonly found in experimental models such as BALB/c, C57Bl/6, and C3H/He/J inbred mice, in which DSS leads to inflammation due to epithelial barrier damage, deregulation of molecular adhesion expression (tight junctions), with consequent dysfunctional intestinal permeability, as reviewed by Eichele and Kharbanda [49]. Importantly, BALB/c, C57BL6, and C3HHeJ mice bear the *Slc11a1 S* allele causative of susceptibility to several infections and may contribute to UC susceptibility in these mouse lines.

The dysfunction of the epithelial barrier and, therefore, increased intestinal permeability caused by DSS, can lead to immune system activation by the microbiota [49, 50].

According to the literature, cellular infiltration in the 7^th^ day DSS phase is predominantly composed of neutrophils and macrophages [51], with macrophages contributing to the production of ROS and inflammatory mediators such as cytokines and chemokines. The cellular infiltrate can affect the integrity of the epithelium, promoting increased permeability with inflammation [9, 51].

These results are in line with some studies have shown that mice lacking T, B, and NK cells can still develop colitis in response to DSS almost independently of lymphocyte actions [52, 53]. No differences in CD4^+^ or CD8^+^ T cell populations were observed between AIRmin^*RR*^ and AIRmin^*SS*^ mice.

In our study we analyzed inflammatory cells such as neutrophils, macrophages, and IL-10-producing macrophages in the colon in association with local cytokine genes expression and cytokines release, aiming to identify factors and possible pathophysiological mechanisms acting in DSS-induced colitis in the two mouse lines.

In these experiments, the macrophage population showed similar profiles in both lines, except for the regulatory IL-10-producing macrophages. That population is considered resident and is characterized by the production of anti-inflammatory cytokines such as IL-10 and TGF-β [54] and is important in the suppression of experimental DSS colitis (together with the resident mucosal dendritic cells) [11]. Thus, the cells with MHC II^+^F4/80^+^IL-10^+^ phenotype found in the colon epithelium after DSS may have a regulatory function in UC, leading to an increase in IL-10 since they were present in ARmin^*RR*^ and not in AIRmin^*SS*^. The pleiotropic effects of the *Slc11a1* gene could affect macrophage activity [26] and contribute to an increase in disease severity in AIRmin^*SS*^ mice.

A strong genetic influence has been observed in human IBD, and polymorphisms of the regulatory cytokine genes (with pro- or anti-inflammatory activities) are associated with susceptibility to IBD and gastrointestinal malignancy and severity [55]. UC in humans is characterized by producing several chemical mediators, such as cytokines, chemokines, and growth factors. Increased levels of IL-1β, for example, are related to inflammatory responses to DSS-induced colitis [49]. Gene expression analyses evidenced an increase in IL-1β in AIRmin^*SS*^ mice, although there was no detection of the protein in the colon lysate. Gene expression of IL-1β increases in the acute phase of UC, with protein levels increasing preferentially in the chronic phase beyond seven days [51, 56].

T lymphocytes, macrophages, and endothelial cells produce IL-6 in the inflamed intestinal mucosa, which contributes to leukocyte influx [57], B cells differentiation [58], antibody-dependent cytotoxicity [59], and hence increased UC severity in both humans and experimental murine models [51, 60]. IL-6 is also involved in signaling and immunoregulatory cascades by binding to its receptor (IL-6R) on gp130-positive cells – thereby activating the transcription factor signal transducer and activators of transcription 3 (STAT3) and increasing the risk of colon cancer [61, 62].

We observed an increase in IL-6 in AIRmin^*SS*^ mice (both mRNA expression and protein synthesis), indicating its importance in the colitis phenotype of those animals. It has been reported that IL-6 levels increased in DSS colitis models in the acute phase of the disease and persisted during the chronic phase [63]. Jiang et al. (2009) also observed an increase in *Il6* gene expression on the 7th day of treatment, although that occurred only in animals with the *Scl11a1*^*wt*^ (wild-type) allele carrying the functional protein [27, 63].

IL-10 is an anti-inflammatory cytokine also present in UC. An increase in the mRNA expression of this cytokine was observed in AIRmin^*SS*^ lines, but there were no significant differences in protein concentrations in their colon lysates. Again, increases in gene expression of that cytokine have been described in DSS models in the acute phase [56] and protein concentrations in the chronic phase of the disease [63]. That could explain the *Il10* gene expression in the AIRmin^*SS*^ group without the presence of the protein in the lysate, probably because it did not reach the chronic stage. Notably, macrophages producing IL-10 were not observed in AIRmin^*SS*^ colon tissue at seven days of DSS treatment.

TNF-α is one of the cytokines considered markers in the DSS colitis model [49], and its gene expression and protein concentrations appear to increase in the acute phase [63]. There were increases in *Tnfα* gene expression in the AIRmin^*RR*^ groups, but they were not accompanied by increased protein concentrations in the colon lysate.

DSS-induced colitis also increases G-CSF growth factor levels [64]. Studies using the DSS colitis model with isogenic lines bearing the mutated G169A *Slc11A.1* allele (C57BL/6 and BALB/c) evidenced an association between G-CSF production and disease chronification [65]. Here, G-CSF increased only in AIRmin^*SS*^ animals, probably related to the destruction of the epithelial barrier by DSS. Kamezaki et al. (2005) showed that, in addition to its role in neutrophil cell maturation and proliferation, G-CSF could also prolong the in vitro life span of those cells by preventing apoptosis during the chronic phase of the disease [66].

In terms of the chemokine MCP-1, Khan et al (2006) demonstrated its importance in the experimental murine model for colitis using DNBS (dinitrobenzenesulfonic acid) for modulating the production of cytokines and inflammatory infiltrate intensity [67]. In our experiments, AIRmin^*SS*^ animals also showed an increase in MCP-1 concentration.

Previous studies about the interference of Slc11a1 polymorphism in DSS-induced colitis pointed out the role of IL6 and IFNγ in this disease [27]. Our data show the participation of other cytokines besides IL-6, such as G-CSF, MCP-1, IL1β, and IL10, which are upregulated during DSS-induced colitis in the susceptible AIRmin SS and not in the resistant AIRmin RR mice. The results suggest that these cytokines might play a role in the pathophysiology of DSS induced Colitis in this mouse model.

Together, our results strongly suggest the influence of the *Slc11a1* gene on the DSS-induced colitis phenotype. The characteristic of colitis resistance exhibited by the parental AIRmin line was altered in AIRmin mice homozygous for the *S* allele that became susceptible, whereas AIRmin^*RR*^ mice remained resistant to severe effects of the drug in the colon.

Previous results have shown that the *Slc11a1 S* allele favored skin tissue repair in AIRmax [68, 69], made AIRmax and AIRmin more susceptible to *Salmonella* Typhimurium infection and more resistant to LPS shock [37] and aggravated the signs of arthritis in AIRmin (resistant) and AIRmax (susceptible) mice [19]. The results demonstrate an interaction between the *Slc11a1* gene and the loci that regulate inflammation, which are involved in these characteristics. The molecular mechanism undermining the significant differences in acute colitis pathology that we have observed between AIRmin^*RR*^ and AIRmin^*SS*^ mice can involve the stimulation of IL-6 and G-CSF production associated with the lack of IL-10-producing cells in the colons following acute inflammatory stress in the large intestine by DSS.

We consider that future comparative studies of transcriptome and microbiota profiling will be necessary to achieve a broader understanding of the regulatory machinery operating in ulcerative colitis sensitivity.

## Supplementary information

Figure S1

## Data Availability

The data used to support the findings of this study are available from the corresponding author upon request.
