# Supplementary material for: Slc11a1 gene polymorphism influences dextran sulfate sodium (DSS)-induced colitis in a murine model of acute inflammation
Source: Genes Immun. 2023 Feb 15;24(2):71–80. doi: 10.1038/s41435-023-00199-7 (PMC10110460; doi:10.1038/s41435-023-00199-7)
Supplement: Supplementary file 1 — Figure S1 [file 41435_2023_199_MOESM1_ESM.docx]

**CD45**

**CD8**


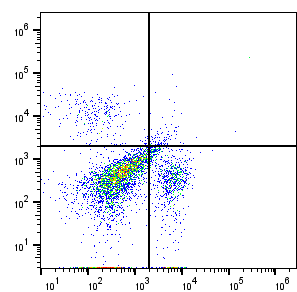


**CD4**

**CD8**


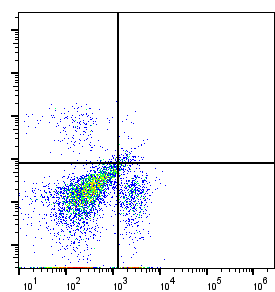

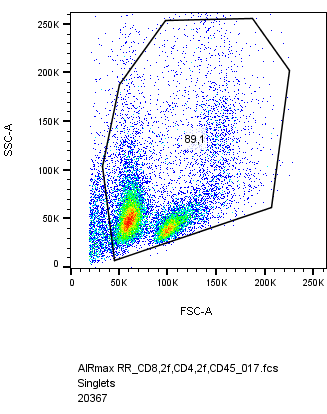

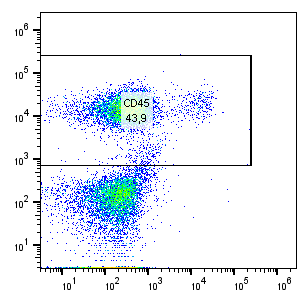


**AIRmin*^SS^***

**AIRmin*^RR^***

**A**

**C**

**B**

**Figure S1:** CD4^+^ and CD8^+^ T cell populations (**A**)-Representative cytograms of the CD4^+^ and CD8^+^ cells from AIRmin*^RR^* and AIRmin*^SS^* mice colon treated with 2.5% DSS (n=5) or that received only water (n=3) AIRmin*^RR^*C and AIRmin*^SS^*C. (**B**) CD4^+^ and (**C**) CD8^+^ cell concentrations. The results are expressed as individual values and averages.
